# Structural and functional retinal alterations in patients with paranoid schizophrenia

**DOI:** 10.1038/s41398-022-02167-7

**Published:** 2022-09-23

**Authors:** Evelyn B. N. Friedel, Hannah-Tabea Hahn, Simon Maier, Sebastian Küchlin, Michael Reich, Kimon Runge, Michael Bach, Sven P. Heinrich, Jürgen Kornmeier, Dominique Endres, Dieter Ebert, Katharina Domschke, Ludger Tebartz van Elst, Kathrin Nickel

**Affiliations:** 1 Department of Psychiatry and Psychotherapy, Medical Center—University of Freiburg, Faculty of Medicine, University of Freiburg, Freiburg, Germany; 2 Eye Center, Medical Center—University of Freiburg, Faculty of Medicine, University of Freiburg, Freiburg, Germany; 3 University of Freiburg, Faculty of Biology, Freiburg, Germany; 4 Augenärzte am Städel, Hans-Thoma-Straße 24, 60596 Frankfurt am Main, Germany; 5 Institute for Frontier Areas of Psychology and Mental Health, Freiburg, Germany; 6 Center for Basics in Neuromodulation, Faculty of Medicine, University of Freiburg, Freiburg, Germany

**Keywords:** Schizophrenia, Neuroscience

## Abstract

Ophthalmological methods have increasingly raised the interest of neuropsychiatric specialists. While the integrity of the retinal cell functions can be evaluated with the electroretinogram (ERG), optical coherence tomography (OCT) allows a structural investigation of retinal layer thicknesses. Previous studies indicate possible functional and structural retinal alterations in patients with schizophrenia. Twenty-five patients with paranoid schizophrenia and 25 healthy controls (HC) matched for age, sex, and smoking status participated in this study. Both, ERG and OCT were applied to obtain further insights into functional and structural retinal alterations. A significantly reduced a-wave amplitude and thickness of the corresponding para- and perifoveal outer nuclear layer (ONL) was detected in patients with paranoid schizophrenia with a positive correlation between both measurement parameters. Amplitude and peak time of the photopic negative response (PhNR) and thickness of the parafoveal ganglion cell layer (GCL) were decreased in patients with schizophrenia compared to HC. Our results show both structural and functional retinal differences between patients with paranoid schizophrenia and HC. We therefore recommend the comprehensive assessment of the visual system of patients with schizophrenia, especially to further investigate the effect of antipsychotic medication, the duration of illness, or other factors such as inflammatory or neurodegenerative processes. Moreover, longitudinal studies are required to investigate whether the functional alterations precede the structural changes.

## Introduction

In recent years, the retina has gained increasing interest as an accessible “window to the brain” for the investigation of psychiatric disorders [1]. The retina is part of the central nervous system and, like the brain, evolves early in development from the anterior part of the neural tube [2]. Previous studies reported impaired visual processing in patients with paranoid schizophrenia. However, the underlying etiology has not been adequately clarified. It is not clear to what extent these visual impairments originate in the brain or the retina [3]. As the diagnosis of paranoid schizophrenia is complex and based on clinical evaluation of positive and negative symptoms, diagnoses supported by objective biomarkers would be desirable [4].

In previous studies, optical coherence tomography (OCT) was applied to gain further insights into structural retinal alterations in patients with schizophrenia [2]. OCT is a noninvasive imaging technique that allows quantification of retinal layer thicknesses and is primarily applied in ophthalmology, e.g., for glaucoma diagnosis [5]. However, it plays an increasing role in the investigation of inflammatory diseases such as multiple sclerosis [6] and neurodegenerative disorders like Parkinson’s [7] and Alzheimer’s disease [8]. The principle of OCT is based on Michelson interferometry [5, 9].

OCT studies in patients with schizophrenia have reported thinning of the peripapillary and/or circumfoveal retinal nerve fiber layer (RNFL) [10–15], the ganglion cell layer (GCL) [12, 14, 16], the macular volume (MV) [10, 11, 15–18], the macular thickness (MT) [10, 11, 13–17, 19, 20], the outer nuclear layer (ONL) [14, 15, 21], and the inner nuclear layer (INL) [15] when compared to healthy controls (HC). Nevertheless, some results point towards normal RNFL thickness in patients with schizophrenia [2, 16–22] as well as normal MT [2, 22]. Sakar et al. [20] detected only reduced MT, but no alterations of the peripapillary RNFL in the early phases of acute schizophrenia and suggested that macular thinning may represent a potential early neurodegeneration marker in schizophrenia.

Lee et al. [10] demonstrated not only a significant thinning but also a negative correlation between the peripapillary RNFL thickness, the MT, and MV with the duration of illness in patients with schizophrenia. Samani et al. [14] reported a negative correlation between the ONL thickness and the severity of negative symptoms. A recent OCT angiography study showed a reduction in retinal microvasculature density in patients with schizophrenia which was associated with macular and peripapillary RNFL thinning and lower verbal IQ scores [23].

While OCT provides information on retinal structure, the electroretinogram (ERG) measures electrophysiological correlates of retinal function [24].

The full-field ERG represents the global electrophysiological response of the retina following flash stimulation and comprises components from the different retinal cells [24, 25]. In the light-adapted ERG, the negative a-wave results from a hyperpolarization of the cone photoreceptors and OFF bipolar cell activation, whereas the positive b-wave represents cone ON and OFF bipolar cell activity [24]. The photopic negative response (PhNR) is a slow negative potential following the b-wave, providing information about ganglion cell function [26, 27].

Studies indicate that these electrophysiological retinal responses might be sensitive to a monoaminergic imbalance, which may explain why ERG investigations have gained interest in psychiatric research [28]. Lavoie et al. [29], for example, reported that alterations in the dopaminergic and serotonergic transmission affect ERG measures in genetically modified mice. Previous ERG studies in Parkinson’s disease showed amplitude reductions in the pattern electroretinogram (PERG) [30], the flash ERG (e.g., reductions in the amplitude of the scotopic a-wave and the photopic b-wave), which was suggested to be likely due to dopaminergic retinal deficiency [31] as well as progressive ERG attenuations in the course of Parkinson’s disease [32]. Likewise, amplitude reductions in the PERG of depressed patients have been observed [33], showing normalization with the remission of depressive symptoms [34]. In a mouse model of Parkinson’s disease (based on a toxin inducing apoptosis within dopaminergic neurons), prolonged peak times of the ERG oscillatory potentials were observed, which were associated with structural thinning of the outer plexiform layer in OCT images [35]. In line, a selective D2 antagonist reduced the steady-state PERG amplitudes and delayed transient PERG responses in healthy participants [36].

In patients with schizophrenia, dysregulation in the different dopaminergic signal pathways is suspected [37, 38]. Recognizing that GABA and dopamine are involved in modulating the activity of retinal horizontal cells during light and dark adaptation, it was suggested that both transmitters should be considered as possible factors for explaining ERG alterations in schizophrenia [39, 40]. Patients with schizophrenia showed ERG modulations like reduced amplitudes of the a-wave [37, 39–43], the b-wave [39, 40, 42], an increased b-wave peak time [39, 42], and PhNR dysfunctions [40, 44].

A current review summarized most consistently reduced amplitudes and prolonged peak times of ERG parameters as well as thinning of the RNFL applied with OCT in schizophrenia [45].

### Aims of the study

The present study aimed to investigate the retinal structure and function of patients with paranoid schizophrenia in comparison to HC by means of OCT and ERG. Both methods were combined to provide comprehensive information on structural and functional retinal alterations in patients with schizophrenia.

The aim was to obtain direct insights into a link between retinal function and the associated retinal layers. Based on previous reports, we expected a thinning of various retinal layers and amplitude reductions of the a- and b-wave and the PhNR in patients with paranoid schizophrenia.

## Material and methods

### Participants

The Ethics Committee of the University Medical Center Freiburg approved the study (Approval ID: 314/18). Investigations were performed in accordance with the Declaration of Helsinki. All participants gave written informed consent.

Twenty-five patients (10 male, 15 female) with paranoid schizophrenia were recruited among outpatients (*N* = 18) and inpatients (*N* = 7) of the Department of Psychiatry and Psychotherapy, University of Freiburg (Table 1). The diagnosis (ICD-10: F20.0) was established according to the criteria of the International Classification of Diseases (ICD-10) by experienced senior specialists in psychiatry and psychotherapy. Patients with schizoaffective disorder were excluded. All but two patients received antipsychotic treatment according to clinical requirements. The severity of positive and negative symptoms was evaluated based on the Positive and Negative Syndrome Scale (PANSS) rating system [46]. Psychological, social, and job-related level of functioning was recorded with the Global Assessment of Functioning (GAF) [47].

**Table 1:** Demographic and psychometric data.

Characteristic	HC *N* = 25	Patients *N* = 25	*p*-value
Sex (female/male)	15/10	15/10	>0.9^a^
Age in years	37 (12)	39 (12)	0.8
Smoking status (smoker/nonsmoker)	13/12	13/12	>0.9^a^
Duration of illness in years		9 (8)	
Medication (unmedicated/medicated)		2/23	
Neuroleptic medication (typical/atypical)		1/23	
CPZ-equivalent dose in mg		355 (271)	
PANSS-total		68 (15)	
PANSS-P		14 (4)	
PANSS-N		18 (5)	
PANSS-G		35 (8)	
CGI-S		4 (1)	
GAF		46 (15)	

The number of observations is depicted for categorial data; numerical data are summarized by the mean and standard deviation (SD).

*CGI* Clinical Global Impression Scale, *CPZ* chlorpromazin, *HC* healthy controls, *GAF* Global Assessment of Functioning, *N* number, *PANSS* Positive and Negative Syndrome Scale, *PANSS-G* Positive and Negative Syndrome Scale—General Psychopathology Scale, *PANSS-N* Positive and Negative Syndrome Scale—Negative Scale, *PANSS-P* Positive and Negative Syndrome Scale—Positive Scale.

Comparisons: Wilcoxon and ^a^Fisher exact tests.

Twenty-five HC (10 male, 15 female) without any previous or current psychiatric disease and without the intake of psychiatric medication were enrolled in the study. HC were evaluated with the SCL-90 questionnaire [48] to exclude psychiatric symptoms and the Beck Depression Inventory (BDI-II) [49, 50] to rule out current depressive episodes. Patients and controls were matched according to sex, age, and smoking status.

Exclusion criteria for both groups were: age <18 or >65 years, neurological or ophthalmological diseases, myopia less than −7 dpt or hyperopia greater than +7 dpt, substance abuse, or diabetes mellitus. Psychiatric medication for the patients or somatic medication for all participants was not defined as an exclusion criterion.

### Data acquisition and processing

#### Electroretinogram (ERG)

**Fig. 1:**
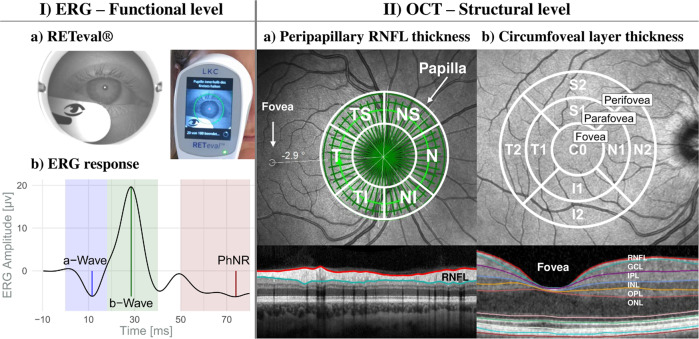
Schematic overview of retinal examinations. (**I**) **ERG:** **a**) Illustration of the RETeval^®^ system during recording (right) and a picture from the device internal camera (left); **b**) Schematic retinal response comprising the a- and b-wave and the PhNR. (**II**) **OCT:** **a**) Peripapillary RNFL thickness from the circle scan with the Garway-Heath-grid; **b**) Circumfoveal scan for layer (macular, RNFL, GCL, IPL, INL, OPL, and ONL) thicknesses and volumes and the ETDRS grid (3 regions: fovea, parafovea, and perifovea). GCL ganglion cell layer, I inferior, INL inner nuclear layer, IPL inner plexifom layer, N nasal, ONL outer nuclear layer, OPL outer plexiform layer, RNFL retinal nerve fiber layer, S superior, T temporal.

Light-adapted ERG examinations for both eyes were carried out with the handheld RETeval^®^ device (LKC Technologies, Inc., version 2.10.2) (Fig. 1), with Sensor-Strip skin electrodes and a protocol where flash strengths compensate for pupil size (Troland modus). After 10 min light adaptation, 200 red flashes (38 Td∙s; 3.4 Hz) were shown on blue background (380 Td) following the recommendations of the International Society for Clinical Electrophysiology of Vision (ISCEV) for PhNR stimulation [27]. The 200 raw responses per eye were collected in one recording session and automatically averaged for the individual ERG-curves (Supplemental Fig. 1A). Data were checked for artifacts, baseline drifts, and correct peak detection prior to the extraction with the RETeval® RFF Extractor software (version 2.9.3.0).

Since previous investigations found reduced ERG amplitudes in people with dark pigmented irises compared to people with blue eyes having lightly pigmented irises [51], we also extracted the iris color index of the HC and patients with schizophrenia with the RETeval® RFF Extractor software. The iris color index is the ratio between the pupil to iris gray values and is automatically calculated based on the videos recorded during the examination. The iris color corresponds to the 25th percentile of the gray values calculated from two 1 mm horizontal line segments, aligned at the left and right edges of the pupil. The 25th percentile of gray values across the horizontal diameter of the pupil (saturated values excluded) corresponds to the pupil color.

Skin electrode placement affects the amplitude of the ERG waveform, with lower amplitudes measured with electrode placement more than 2 mm below the lower eyelid [52]. Therefore, we measured the distances between the upper edge of the electrode and the lower eyelid using ImageJ (version: 1.53k) based on the pictures extracted with the RETeval® RFF Extractor software.

The a-wave amplitude was measured from the pre-stimulus baseline to the first trough (Fig. 1). The b-wave amplitude was defined as the difference between the a-wave minimum and the following maximum positive deflection. PhNR was measured relative to the pre-stimulus baseline as the maximum negative deflection within the 55–100 ms post-stimulus interval and additionally at 72 ms, as suggested by Preiser et al. [53]. Moreover, both PhNR-ratios were extracted, the P-ratio defined as: PhNR at 72 ms / b-wave [53] and the W-ratio as: (b-wave − PhNR) / (b-wave − a-wave) [54].

All peak parameters (peak amplitude and time of the a-wave, b-wave, and PhNR) were averaged across both individual eyes after ensuring proper intercorrelation coefficients (ICC).

#### Optical coherence tomography (OCT)

Retinal imaging for both eyes was conducted using the SPECTRALIS® OCT (Heidelberg Engineering; 880 nm mean wavelength of the superluminescent diode) and the Glaucoma Module Premium Edition® (Acquisition Software version 6.9.4.0) with the Eye Explorer® (version 1.10.2.0). A-scans were sampled with 40 kHz in 3.9 µm/pixel depth resolution. The Anatomic Positioning System was used for automated papillae and fovea detection.

1. The peripapillary RNFL thickness was measured with the “optic nerve head—radial circle scan”. During nasal fixation, 768 A-scans and 27 B-scans (24 radial scans for Bruch’s membrane opening (BMO) detection and 3 circle scans with 3.5, 4.1, 4.7 mm diameter) were made within a 15° scan angle around the BMO center. Analysis of the peripapillary RNFL was restricted to the innermost circular scan (3.5 mm). The 6-sector Garway-Heath grid was used for analysis (Fig. 1) [55].
2. A “posterior pole horizontal scan” was applied during central fixation. Seven hundred sixty-eight A-scans and sixty-one B-scans were taken within a field of 30° × 25° (121 µm distance) around the fovea. Layer thickness and MV were evaluated using the 9-sector grid from the Early Treatment Diabetic Retinopathy Study (ETDRS) [56] (Fig. 1). The grid sectors were pooled into three regions: fovea (C0; 1 mm), parafovea (S1, N1, I1, T1; 3 mm) and perifovea (S2, N2, I2, T2; 6 mm).

OCT images were checked for artifacts or incorrect layer segmentation and analyzed by an ophthalmologist for pathological findings before data extraction.

### Statistical analysis

Statistical analysis was performed in “R” [57] with RStudio [58] using the “tidyverse” package [59]. If available, data from both eyes were averaged for all participants.

Since the normal distribution was not given in all data records, nonparametric Wilcoxon Tests (“rstatix” package [60]) were performed for group comparisons between HC and patients. The significance level was defined as α = 0.05 and adjusted according to the Bonferroni–Holm procedure [61]. Cohen’s *d* (“rstatix” package [62]) was calculated as an effect size estimation [63].

Explorative Spearman correlation coefficients were calculated (“correlation” package [64]) to evaluate associations between ERG and OCT data, chlorpromazine equivalent doses (CPZ-doses [65]), the duration of illness, and PANSS scores.

#### ERG

One-sided Wilcoxon tests were conducted with the assumption of smaller peak amplitudes in the patient group when compared to HC. No assumptions were made for peak times and PhNR-ratios. The significance level was adjusted according to the number of peak components. Additional comparisons between male and female HC and patients with schizophrenia were performed.

#### OCT

Circumfoveal OCT data were evaluated stepwise:

1. Total ETDRS volume and thickness of all retinal layers (MT/MV, RNFL, GCL, IPL, INL, OPL, and ONL) were compared between groups adjusting the significance level according to the number of layers.
2. Region-specific group differences were evaluated per layer for the three regions (fovea, peri-, and parafovea) of the ETDRS grid. Results were corrected with respect to the number of regions per layer.
3. Since the anatomical directions (superior, nasal, inferior, and temporal) cannot be considered within the peri- and parafoveal rings, we finally compared the sector thicknesses between groups. The number of sectors per region was considered for Bonferroni–Holm adjustment.

#### Comparison of functional (ERG) and structural (OCT) data

Spearman’ *rho* was calculated to compare ERG amplitudes and layer thicknesses separately for HC and patients. Calculations were limited to combinations of ERG components and their corresponding structure of origin. The a-wave was correlated to the most distal layers (INL, OPL, and ONL), while the PhNR was compared to the most proximal layers (RNFL and GCL). The b-wave amplitude was compared to the IPL, INL, and OPL.

## Results

### Demographic and psychometric data

Table 1 illustrates the demographic and psychometric data of all participants. Twenty-five patients with paranoid schizophrenia (ICD-10: F20.0) were investigated. In addition, 25 HC matched for sex, age, and smoking status were included.

Except for two unmedicated individuals, all patients took neuroleptic medication (23 atypical, one atypical in combination with a typical antipsychotic medication). Eight patients were treated with additional antidepressants or mood stabilizers (five SSRIs, one combined with valproate and lithium, two took SSNRIs, one in combination with trimipramine, and one received valproat). Another patient took biperiden and zopiclone in addition to the antipsychotic medication.

### Ophthalmological evaluation and exclusion

Six eyes in the patient and three eyes in the HC group were excluded due to unrelated pathologic findings on OCT scans, incorrect data acquisition, or excessive artifacts. This yielded a set of 44 eyes of 25 patients with schizophrenia and 47 eyes of 25 HC for final analysis. One patient did not tolerate the photopic stimulation for the ERG recording, so only 24 patients took part in the ERG measures.

Intercorrelation coefficients (ICC) for the ERG peak amplitudes of all participants showed good consistency between both eyes (Supplemental Fig. 2).

The vertical position of the electrode did not differ significantly between HC and patients with schizophrenia (right eye (*p* = 0.51): HC mean (SD): 1.3 (0.54) mm, range: 0.51–2.45 mm; patients with schizophrenia mean (SD): 1.38 (0.51) mm, range: 0.78–3.07 mm; left eye (*p* = 0.09): HC mean (SD): 1.35 (0.59) mm, range: 0.58–2.57 mm; patients with schizophrenia mean (SD): 1.05 (0.38) mm, range: 0.59–2.06 mm).

Both groups did not differ in the iris color index (HC mean (SD): 1.16 (0.04), range: 1.09–1.27; patients with schizophrenia mean (SD): 1.20 (0.09), range: 1.08–1.41; *p* = 0.25).

### Flash electroretinogram (ERG)

**Fig. 2:**
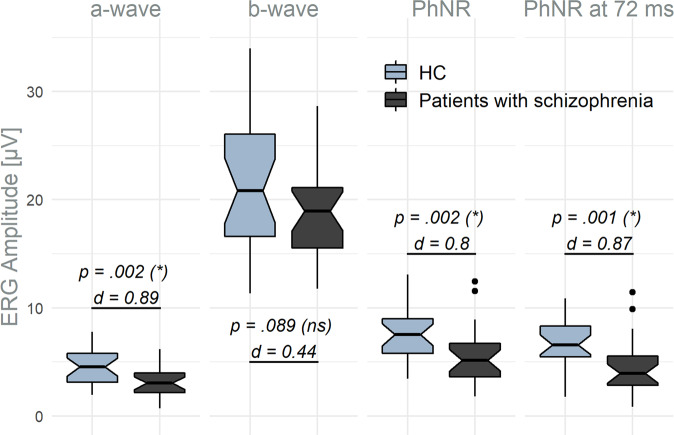
ERG amplitudes (μV) for HC and patients with schizophrenia. *P*-values of the one-sided Wilcoxon tests with Bonferroni–Holm-corrected significance levels in parentheses and Cohen’s d (*d*). *d* Cohen’s d, HC healthy controls, ns not significant, * significant.

Figure 2 depicts the amplitudes of the a- and b-wave, the PhNR, and the PhNR at 72 ms for both patients and HC. Patients with paranoid schizophrenia showed a significantly reduced a-wave and PhNR (in both measures) as well as a smaller P-ratio (mean (SD); HC: 0.43 (0.17); patients: 0.30 (0.16); *p* = 0.006, *d* = 0.78) in comparison to HC (Supplemental Table 1).

Two-sided Wilcoxon tests for peak times revealed an earlier PhNR component (mean (SD); HC: 72.7 (5.2) ms; patients: 68.1 (7.1) ms; *p* = 0.013, *d* = 0.74) in patients with schizophrenia when compared to HC, while no differences in peak time of the a-wave (*p* = 0.63) and b-wave (*p* = 0.39) were detected (Supplemental Table 1).

No differences between female and male HC or female and male patients with schizophrenia were found for all investigated ERG parameters (Supplemental Table 2, Supplemental Fig. 1B).

### Optical coherence tomography (OCT)

#### Peripapillary RNFL thickness

No significant group differences in the peripapillary RNFL thickness were detected.

#### Circumfoveal layer analysis

##### ETDRS total thickness and volume

One-sided Wilcoxon tests showed a significant thinning in the ONL total thickness (mean (SD) in µm; HC: 640.9 (48.3) vs. patients: 597.8 (78.2); *p* = 0.005, *d* = 0.66) and volume (mean (SD) in mm³; HC: 1.8 (0.1) vs. patients: 1.7 (0.2); *p* = 0.003, *d* = 0.73).

No differences in total macular, GCL, RNFL, IPL, INL, or OPL thicknesses or volumes were found between HC and patients after Bonferroni–Holm adjustment.

##### Foveal regions within the ETDRS grid

**Fig. 3:**
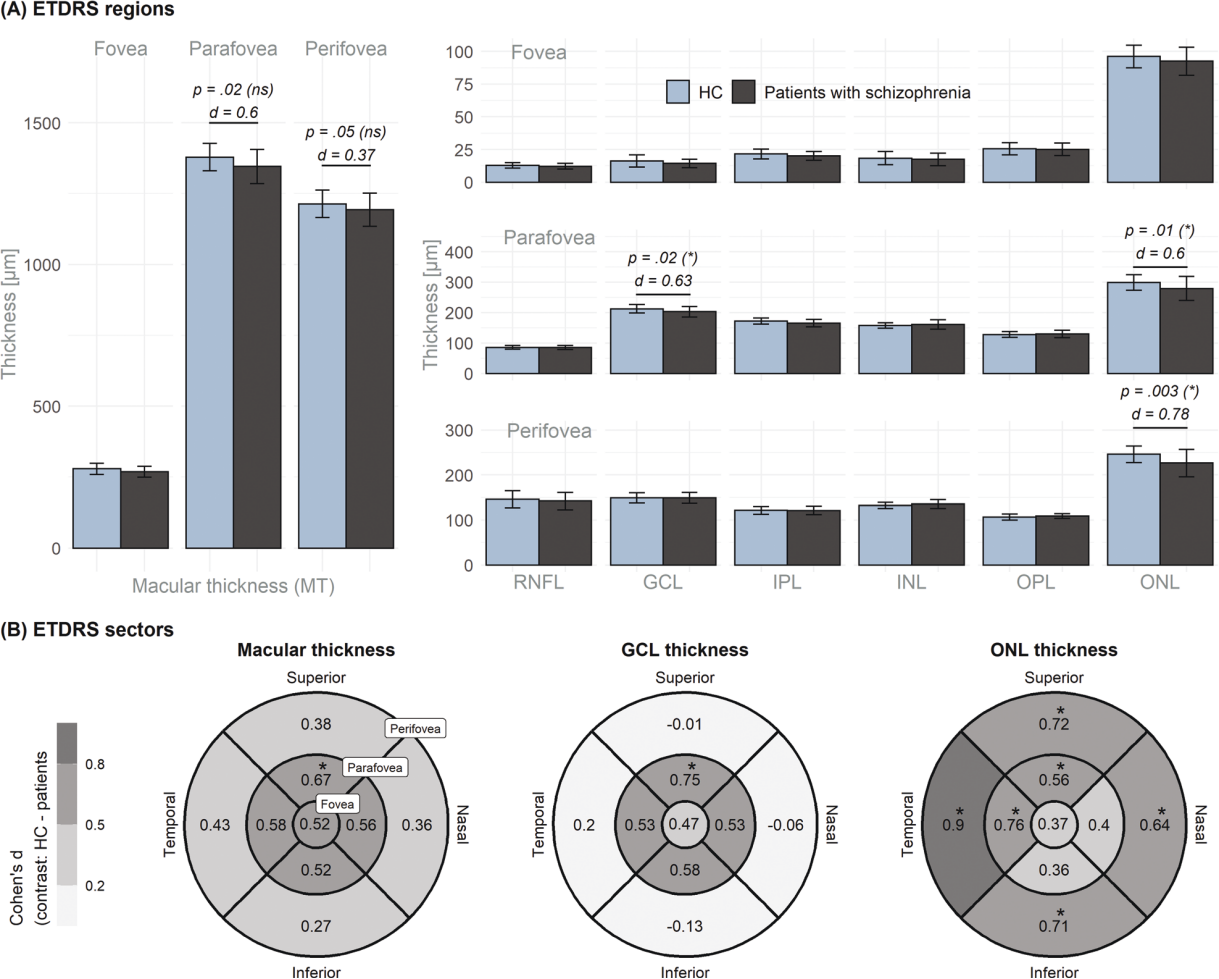
Circumfoveal layer thickness. **A** The foveal regions of the ETDRS grid. *P*-values < 0.05 from tests for the macular, GCL, and ONL thicknesses; Bonferroni–Holm adjusted significance level in parentheses. **B** ETDRS sector analysis for the macular, GCL, and ONL thicknesses. Cohen’s d (*d*) contrasting HC and patients. Significant group differences are indicated by the “*”. The number of sectors in the para- and perifoveal rings were considered for Bonferroni–Holm adjustment. *d* Cohen’s d, GCL ganglion cell layer, HC healthy controls, INL inner nuclear layer, IPL inner plexifom layer, ns not significant, ONL outer nuclear layer, OPL outer plexiform layer, RNFL retinal nerve fiber layer, * significant.

Figure 3A depicts the layer thicknesses on a regional level with the results from the one-sided Wilcoxon analysis included (Supplemental Table 3). Significant thinning of the peri- and parafoveal ONL and the parafoveal GCL in patients with paranoid schizophrenia were observed.

##### Group comparisons on EDTRS sector level

Figure 3B depicts the effect sizes for group differences in the macular, GCL, and ONL thicknesses within the ETDRS grid (Cohen’s *d* for the 9-sector tiles). A significant thinning in patients with schizophrenia was observed in the superior parafoveal MT and GCL. ONL thinning was observed in the superior and temporal parafoveal sectors and in all sectors of the outer perifoveal ring (Supplemental Table 3).

### Correlation between ERG and OCT data with psychometric and demographic data

For the patients with schizophrenia, no significant correlations between the ERG peak amplitudes (a-wave, PhNR) or OCT thickness data (macular, GCL, or ONL thickness) with the CPZ-doses, the duration of illness, or the PANSS scores were indicated after correction for multiple testing.

### Exploratory correlation analysis between functional (ERG) and structural (OCT) data

**Fig. 4:**
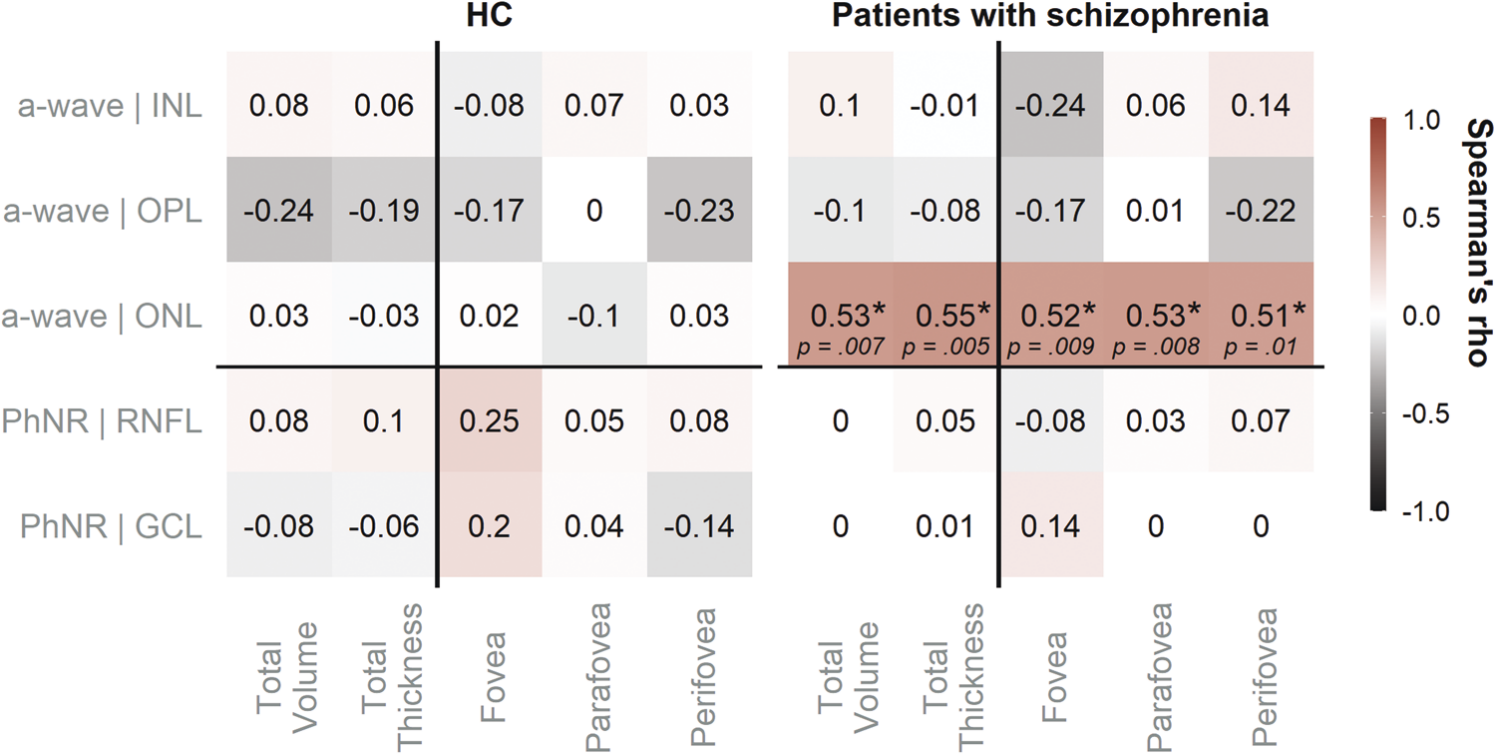
Exploratory correlation analysis. Spearman’s *rho* (depicted) computed for correlation analyses comparing ERG amplitudes and thicknesses of the corresponding layers of origin. Significant correlations (uncorrected) are marked by “*”. HC healthy controls, GCL ganglion cell layer, INL inner nuclear layer, IPL inner plexifom layer, ONL outer nuclear layer, OPL outer plexiform layer, RNFL retinal nerve fiber layer, * significant.

Correlation analysis revealed no significant associations between ERG and OCT data in the HC group. For the patients with schizophrenia, comparisons were limited to a-wave and PhNR amplitudes with their corresponding structures of origin (Fig. 4).

In the patient group significant correlations were observed between the a-wave amplitude and the ONL total volume (*rho* = 0.53, *p* = 0.007), total thickness (*rho* = 0.55, *p* = 0.005) and with the thicknesses from all foveal regions (fovea: *rho* = 0.52, *p* = 0.009; parafovea: *rho* = 0.53, *p* = 0.008 and perifovea: *rho* = 0.51, *p* = 0.01). No other significant correlations were detected for the a-wave and PhNR amplitudes with the thicknesses of their possible structures of origin.

## Discussion

To obtain more detailed insights into visual alterations in schizophrenia, the retina of patients with schizophrenia was assessed functionally by ERG and structurally by OCT.

Attenuation of the a-wave, the PhNR, and an earlier PhNR response were observed in patients with schizophrenia compared to HC. Areas in the peri- and parafoveal ONL and parts of the parafoveal GCL showed a thinning in patients with schizophrenia.

The reduced a-wave reflecting photoreceptor dysfunction in schizophrenia is consistent with previous studies [37, 39–43]. We can also confirm a thinning in the ONL [14, 15, 21], containing the photoreceptor cell bodies, of patients with schizophrenia.

While Schönfeldt-Lecuona et al. [15] described the perifoveal ONL thickness of the right eye measures to be reduced, Samani et al. [14] and Bannai et al. [21] reported thinning in the foveal and parafoveal ONL. They suggested that this might reflect photoreceptor cell loss causing downstream modulations of visual signals manifesting in abnormal ERG a-waves [21].

Our results not only confirm that both para- and perifoveal regions of the ONL are thinned in patients with schizophrenia, but also reveal associations between the ERG a-wave amplitude and the thickness of the ONL as manifest in the significant positive correlations between both measures in the patient group.

Moreover, Bannai et al. [21] discussed a predictive property of the ONL with regard to cognitive performance, since they found positive correlations between the ONL thickness not only with the whole brain and white matter volume but also with the psychometric data of the Brief Assessment of Cognition in Schizophrenia instrument.

Concordant with preceding investigations, we found thinning of the GCL—encompassing the ganglion cell bodies [12]—in patients with schizophrenia [12, 14, 16]. Samani et al. [14] showed temporal parafoveal GCL thinning and suggested that the loss of ganglion cells may be due to altered dopamine-mediated connections with amacrine cells [66]. Celik et al. [12] reported that the GCL volumes in treatment-resistant patients, with a high percentage of clozapine use, were significantly smaller than those of the treatment-responsive patients.

Our result of an unaltered b-wave amplitude in schizophrenia is consistent with some earlier studies with small sample sizes [37, 41], but in contrast to several investigations in larger samples which showed a reduced b-wave amplitude [39, 40, 42]. This highlights the importance of studying larger samples in the future. Despite some studies pointing towards delayed b-wave peak times in patients with schizophrenia [39, 40, 42], others reported unaltered timing of the b-wave [43, 44], consistent with our results.

Moreover, the finding of an unaltered a-wave peak time in the current study is in concordance with a large number of previous investigations [37, 39–41, 44].

With regard to the PhNR our results support the findings of Demmin et al. [40] reporting PhNR attenuations in patients with schizophrenia, but are in contrast to the results of Moghimi et al. [44] describing elevated PhNR amplitudes at high flash strengths and increased response variability in males. In contrast, we observed no significant differences in the ERG peak amplitudes, times, and PhNR-ratios between males and females or an increased response variability in males. Compared to Moghimi et al. [44] applying a flash strength of 420 Td∙s, we chose a flash strength of 37 Td∙s, which is recommended by the ISCEV for stimulation of the PhNR.

Although the PhNR mainly originates from both the retinal ganglion cell bodies and their axons [26, 27] and our patients with schizophrenia showed an attenuation of the PhNR along with a thinning in the GCL, the functional and structural changes seem to occur independently since no correlation was observed.

Antipsychotic medication can lead to a blockade of retinal dopamine receptors, which is suggested to cause ganglion cell death resulting in retinal thinning [2, 3]. A negative correlation between the CPZ-dose and the RNFL thickness was demonstrated in an earlier study [16], whereas the effects of antipsychotic medication on ERG parameters are questioned [39, 40].

Our results indicate retinal differences between patients with schizophrenia and HC. There is thus an urgent need to further investigate the underlying differences to clarify whether, for example, inflammatory processes, antipsychotic medication, or the course of the disease contributes to structural thinning [11] in patients with paranoid schizophrenia.

Finally, it remains unresolved whether functional loss precedes structural retinal changes, as described for optic nerve atrophy [67], or whether the alterations in the PhNR and the GCL thickness do not share the same etiology. For instance, the PhNR could represent the intrinsic dopaminergic state, while ganglion cell loss might be attributed to antipsychotic medication.

It is also conceivable that retinal thinning and impaired function may reflect neuronal degeneration associated with the disease, or even represent some kind of predisposing marker.

Longitudinal studies focusing on medication effects, psychotic status, and the investigation of unmedicated patients with schizophrenia as well as patients at increased risk for psychotic symptoms are desirable. This could clarify whether the structural changes occur independent of dopaminergic treatment and acute phases, thus representing a potential trait marker for schizophrenia.

Even though additional studies are required to pinpoint the origin of retinal pathology in patients with schizophrenia, the comprehensive assessment of the visual system of patients with schizophrenia may offer the potential as an objective marker for the disease, supporting the monitoring of medical treatment.

### Methodological issues and limitations

The sample size limits the results as test strength is possibly underpowered. However, the sample size for both groups is comparable to former studies. While previous studies reported no influence of medication on ERG measures [39, 40], a confounding effect of the antipsychotic treatment cannot be excluded. So far, smoking has rarely been considered a confounding factor [21], despite reports of negative correlations between smoking and retinal thickness [68] exist. While patients and controls in this study were matched by smoking status, a consideration of the daily amount of nicotine intake might have provided further robustness.

### Summary

At the structural level, we detected thickness reductions in patients with schizophrenia in the peri- and parafoveal ONL and the parafoveal GCL when compared to HC. At the functional level, decreased a-wave and PhNR amplitudes were observed in patients with schizophrenia. For the patients, we found positive correlations between the a-wave amplitude and the ONL thickness.

## Supplementary information

Supplemetal Material

Figure SF1

Figure SF2

## Data Availability

The datasets and “R” codes of the current study are available from the first (EF) and corresponding author (KN).
