# Supplementary material for: Structural and functional retinal alterations in patients with paranoid schizophrenia
Source: Transl Psychiatry. 2022 Sep 23;12:402. doi: 10.1038/s41398-022-02167-7 (PMC9508100; doi:10.1038/s41398-022-02167-7)
Supplement: Supplementary file 1 — Supplemetal Material [file 41398_2022_2167_MOESM1_ESM.docx]

**Supplementary Material**

**Supplemental Tables (ST)**

**Table ST1: ERG results comparing HC and patients with schizophrenia.** The mean and standard deviation (SD) for the ERG peak amplitudes in µV and peak times in ms as well as PhNR-ratios are summarised. P-values from the Wilcoxon tests, the Bonferroni-Holm adjusted level of significance in parentheses and Cohen’s d (*d*) are depicted. PhNR-ratios were calculated based on the peak amplitudes relative to the pre-stimulus baseline. The P-ratio is defined as: PhNR at 72 ms / b-wave, the W-ratio is defined as: (b-wave – PhNR) / (b-wave – a-wave).

| Parameter | Peak | HC N = 25 | Patients N = 24 | *p*-value | *d* |
| --- | --- | --- | --- | --- | --- |
| Peak amplitude [µV] | a-wave | 4.6 (1.7) | 3.1 (1.5) | .002 (*) | 0.89 |
|  | b-wave | 21.6 (6.1) | 19.2 (4.6) | .089 (ns) | 0.44 |
|  | PhNR | 7.6 (2.4) | 5.6 (2.7) | .002 (*) | 0.80 |
|  | PhNR at 72 ms | 6.8 (2.3) | 4.6 (2.6) | .001 (*) | 0.87 |
| Peak time [ms] | a-wave | 11.9 (0.9) | 11.7 (0.7) | .630 (ns) | 0.15 |
|  | b-wave | 28 (1.4) | 28.4 (1.9) | .390 (ns) | -0.23 |
|  | PhNR | 72.7 (5.2) | 68.1 (7.1) | .013 (*) | 0.74 |
| PhNR-Ratio | P-ratio | 0.43 (0.17) | 0.30 (0.16) | .006 (*) | 0.78 |
|  | W-ratio | 1.16 (0.14) | 1.13 (0.13) | .532 (ns) | 0.21 |

Abbreviations: *d* = Cohen’s d; ns = not significant; PhNR = photopic negative response; P-ratio = PhNR at 72 ms/ b-wave; SD = standard deviation; W-ratio = (b-wave – PhNR) / (b-wave – a-wave); * = significant.

**Table ST2: ERG results comparing female and male participants within both groups** (HC: 15 ♀ and 10 ♂; patients with schizophrenia: 14 ♀ and 10 ♂). The mean, the standard deviation (SD) and the standard error (SE) for the ERG peak amplitudes, peak times and PhNR-ratios are summarised. P-values from two-sided Wilcoxon tests and the level of significance in parentheses are depicted. PhNR-ratios were calculated based on the peak amplitudes relative to the pre-stimulus baseline. The P-ratio is defined as: PhNR at 72 ms / b-wave, the W-ratio is defined as: (b-wave – PhNR) / (b-wave – a-wave).

| Parameter | Peak | Group | Female | | Male | | *p*-value |
| --- | --- | --- | --- | --- | --- | --- | --- |
|  |  |  | Mean | SD / SE | Mean | SD / SE |  |
| Peak amplitude [µV] | a-wave | HC | 5.1 | 1.6 / 0.4 | 3.7 | 1.6 / 0.5 | .08 (ns) |
|  |  | Patients | 3.1 | 1.7 / 0.5 | 3.2 | 1.2 / 0.4 | .89 (ns) |
|  | b-wave | HC | 23.1 | 4.3 / 1.1 | 19.3 | 7.7 / 2.4 | .07 (ns) |
|  |  | Patients | 19.1 | 4.9 / 1.3 | 19.4 | 4.5 / 1.4 | .98 (ns) |
|  | PhNR | HC | 8.2 | 2.3 / 0.6 | 6.7 | 2.3 / 0.7 | .22 (ns) |
|  |  | Patients | 6.0 | 2.6 / 0.7 | 5.0 | 2.9 / 0.9 | .21 (ns) |
|  | PhNR  at 72 ms | HC | 7.3 | 2.4 / 0.6 | 6.0 | 2.0 / 0.6 | .09 (ns) |
|  |  | Patients | 5.0 | 2.7 / 0.7 | 4.2 | 2.5 / 0.8 | .37 (ns) |
| Peak time [ms] | a-wave | HC | 11.8 | 0.9 / 0.2 | 11.9 | 1.0 / 0.3 | .80 (ns) |
|  |  | Patients | 11.6 | 0.9 / 0.2 | 11.9 | 0.5 / 0.1 | .30 (ns) |
|  | b-wave | HC | 27.8 | 1.2 / 0.3 | 28.3 | 1.8 / 0.6 | .60 (ns) |
|  |  | Patients | 27.9 | 2.0 / 0.5 | 29.0 | 1.8 / 0.6 | .10 (ns) |
|  | PhNR | HC | 73.7 | 3.7 / 1.0 | 71.2 | 6.9 / 2.2 | .40 (ns) |
|  |  | Patients | 66.9 | 6.1 / 1.6 | 69.7 | 8.4 / 2.7 | .30 (ns) |
| PhNR-ratio | P-ratio | HC | 0.42 | 0.14 / 0.04 | 0.44 | 0.23 / 0.07 | .81 (ns) |
|  |  | Patients | 0.31 | 0.14 / 0.04 | 0.27 | 0.19 / 0.06 | .40 (ns) |
|  | W-ratio | HC | 1.14 | 0.09 / 0.02 | 1.20 | 0.20 / 0.06 | .81 (ns) |
|  |  | Patients | 1.16 | 0.14 / 0.04 | 1.10 | 0.12 / 0.04 | .23 (ns) |

Abbreviations: HC = healthy controls; ns = not significant; PhNR = photopic negative response; P-ratio =  PhNR at 72 ms / b-wave; SD = standard deviation; SE = standard error; W-ratio = (b-wave – PhNR) / (b-wave – a-wave); ♀ = female; ♂ = male.

**Table ST3: OCT results from the post hoc analysis on region and sector level.** The mean and standard deviation (SD) for the three regions and the ETDRS sectors of the macular, GCL and ONL thicknesses in µm are shown. *P-*values from the one-sided Wilcoxon tests, the level of significance after Bonferroni-Holm adjustment and Cohen’s d (*d*) is depicted.

| Layer | Region | HC | Patients | *p*-value | *d* |
| --- | --- | --- | --- | --- | --- |
| MT | Fovea | 278.5 (19.5) | 268.4 (19.6) | .056 (ns) | 0.52 |
|  | Parafovea | 1378.8 (48.5) | 1346.1 (60.3) | .017 (ns) | 0.60 |
|  | Perifovea | 1213.7 (48.4) | 1193.6 (58.6) | .046 (ns) | 0.37 |
| GCL | Fovea | 16.2 (4.7) | 14.3 (3.3) | .11 (ns) | 0.47 |
|  | Parafovea | 212.5 (14.2) | 202.6 (17.1) | **.016 (*)** | 0.63 |
|  | Perifovea | 149.1 (11.4) | 148.9 (11.8) | .38 (ns) | 0.02 |
| ONL | Fovea | 96.1 (8.6) | 92.5 (10.7) | .12 (ns) | 0.37 |
|  | Parafovea | 298.7 (25.2) | 278.9 (39.4) | **.01 (*)** | 0.60 |
|  | Perifovea | 246.1 (18.5) | 226.4 (30.6) | **.003 (*)** | 0.78 |
| **Layer** | **Sector** | **HC** | **Patients** | ***p*-value** | ***d*** |
| MT | S1 | 349.8 (12.8) | 340.5 (14.8) | **.01 (*)** | 0.67 |
| GCL | S1 | 349.8 (12.8) | 340.5 (14.8) | **.01 (*)** | 0.67 |
| ONL | S1 | 55.1 (3.4) | 52.4 (3.7) | **.006 (*)** | 0.75 |
|  | S1 | 75.1 (9.4) | 68.9 (12.4) | **.014 (*)** | 0.56 |
|  | S2 | 66 (6.1) | 60.5 (8.8) | **.005 (*)** | 0.72 |
|  | N2 | 62.7 (5.8) | 57.9 (8.7) | **.007 (*)** | 0.64 |
|  | I2 | 56.3 (3.8) | 52.2 (7.1) | **.008 (*)** | 0.71 |
|  | T1 | 75.7 (6.3) | 69.7 (9.2) | **.002 (*)** | 0.76 |
|  | T2 | 61.2 (4.1) | 55.7 (7.5) | **.001 (*)** | 0.90 |

Abbreviations: *d* = Cohen’s d; GCL = ganglion cell layer; I = inferior; MT = macular thickness; N = nasal; ns = not significant; ONL = outer nuclear layer; S = superior; SD = standard deviation; T = temporal; * = significant.

**Supplemental Figures (SF)**

**Figure SF1: Individual ERG traces** for HC and patients with schizophrenia (SF1A) and female and male HC (15 ♀; 10 ♂) and patients with schizophrenia (14 ♀; 10 ♂) respectively (SF1B). The mean ERG responses (bold lines) and standard deviations (shaded ribbons) are illustrated. Flash onset is marked with a solid vertical line (0 ms), a long-dashed vertical line indicates the PhNR at 72 ms post stimulus.


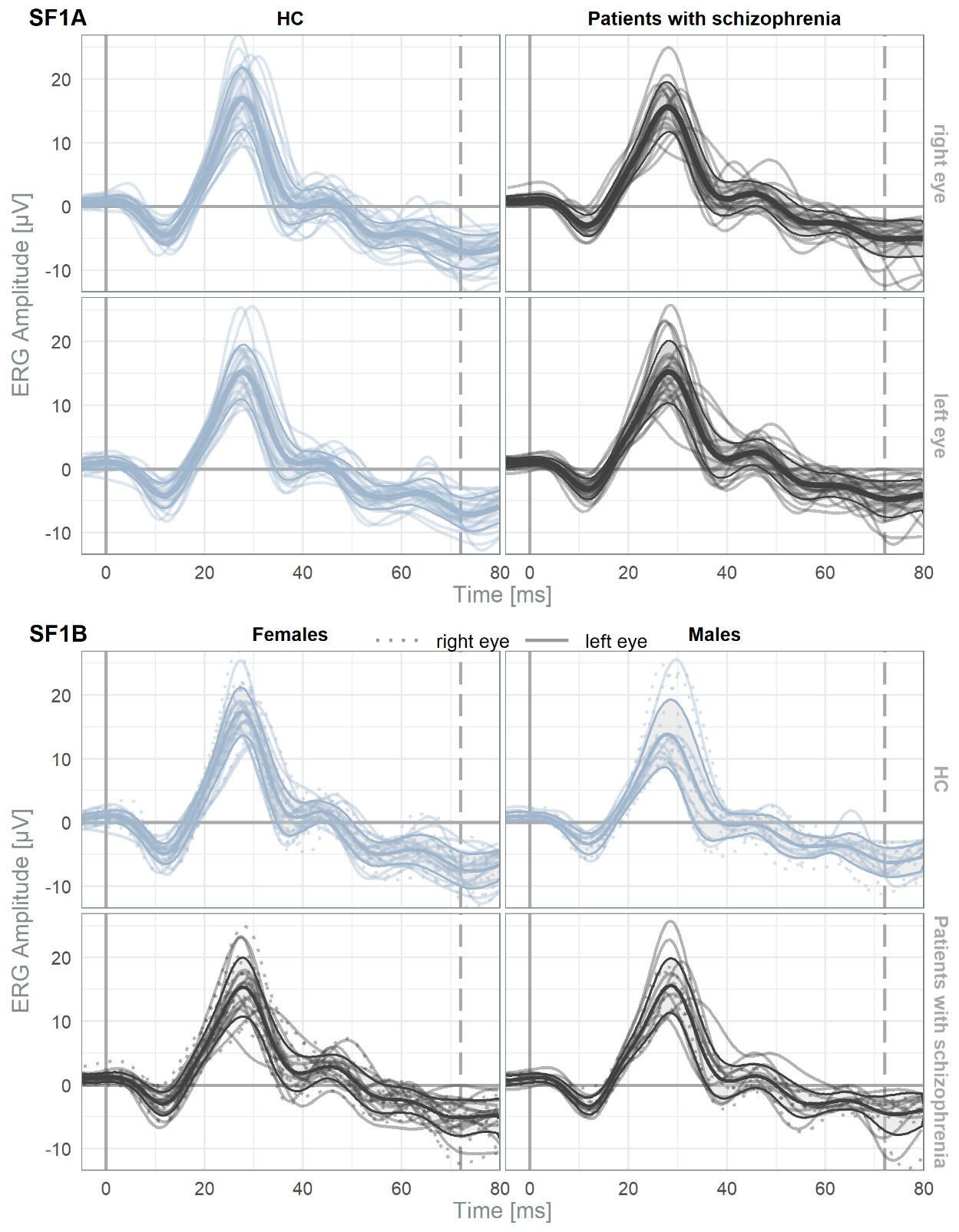


Abbreviations: ERG = electroretinogram; HC = healthy controls; PhNR = photopic negative response; ♀ = female; ♂ = male.

**Figure SF2:** **Peak amplitudes in µV of both eyes and all ERG components with the corresponding intercorrelation coefficients (ICC) calculated for all participants.** ICC thresholds: ICC < 0.5 = poor, ICC ≥ 0.5 and < 0.75 = moderate, ICC ≥ 0.75 and < 0.90 = good and ICC values ≥ 0.90 = excellent.


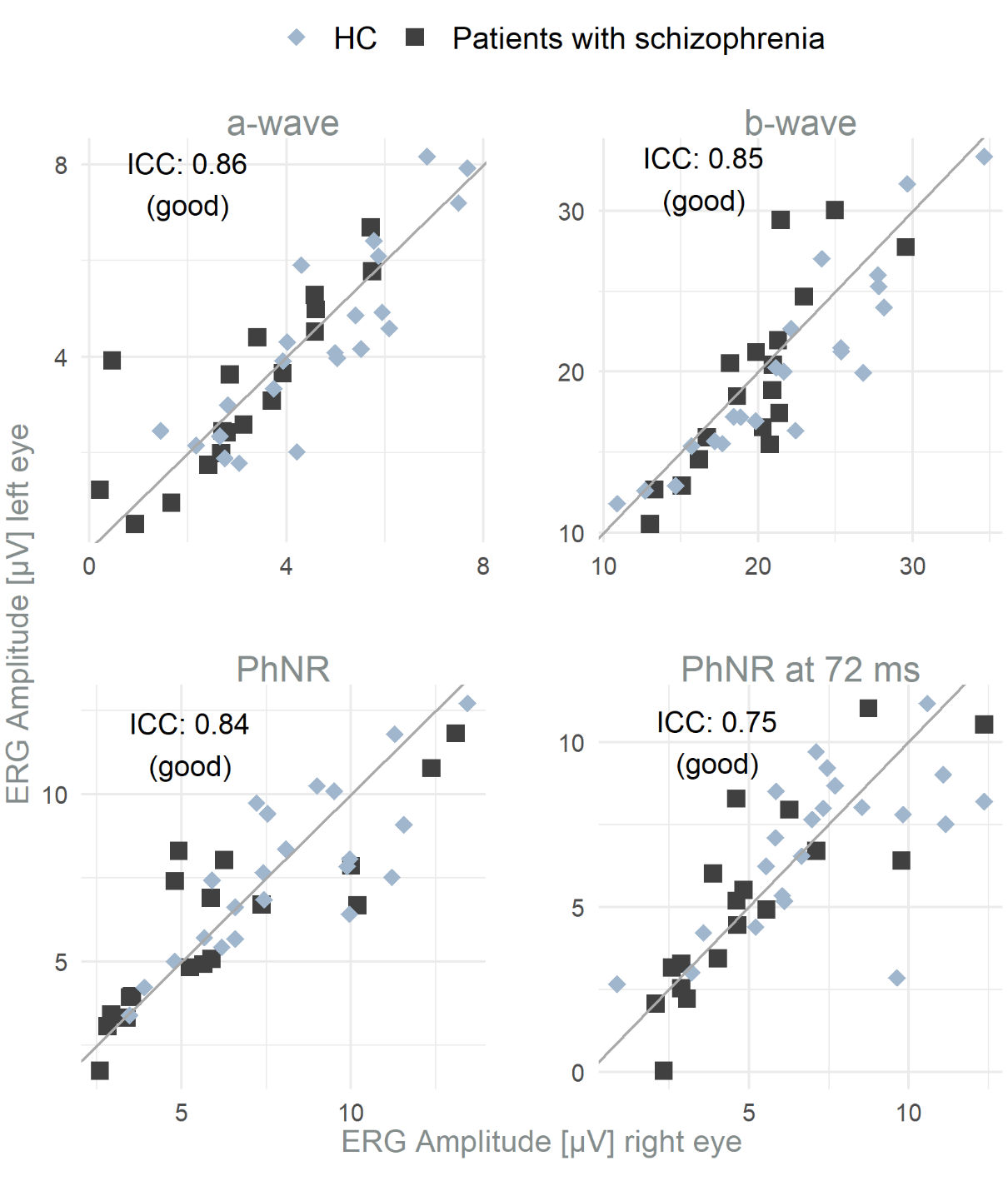


Abbreviations: ERG = electroretinogram; HC = healthy controls; ICC = intercorrelation coefficient; PhNR = photopic negative response.
