# Supplementary figures and images for: Structural and functional retinal alterations in patients with paranoid schizophrenia
Source: Transl Psychiatry. 2022 Sep 23;12:402. doi: 10.1038/s41398-022-02167-7 (PMC9508100; doi:10.1038/s41398-022-02167-7)

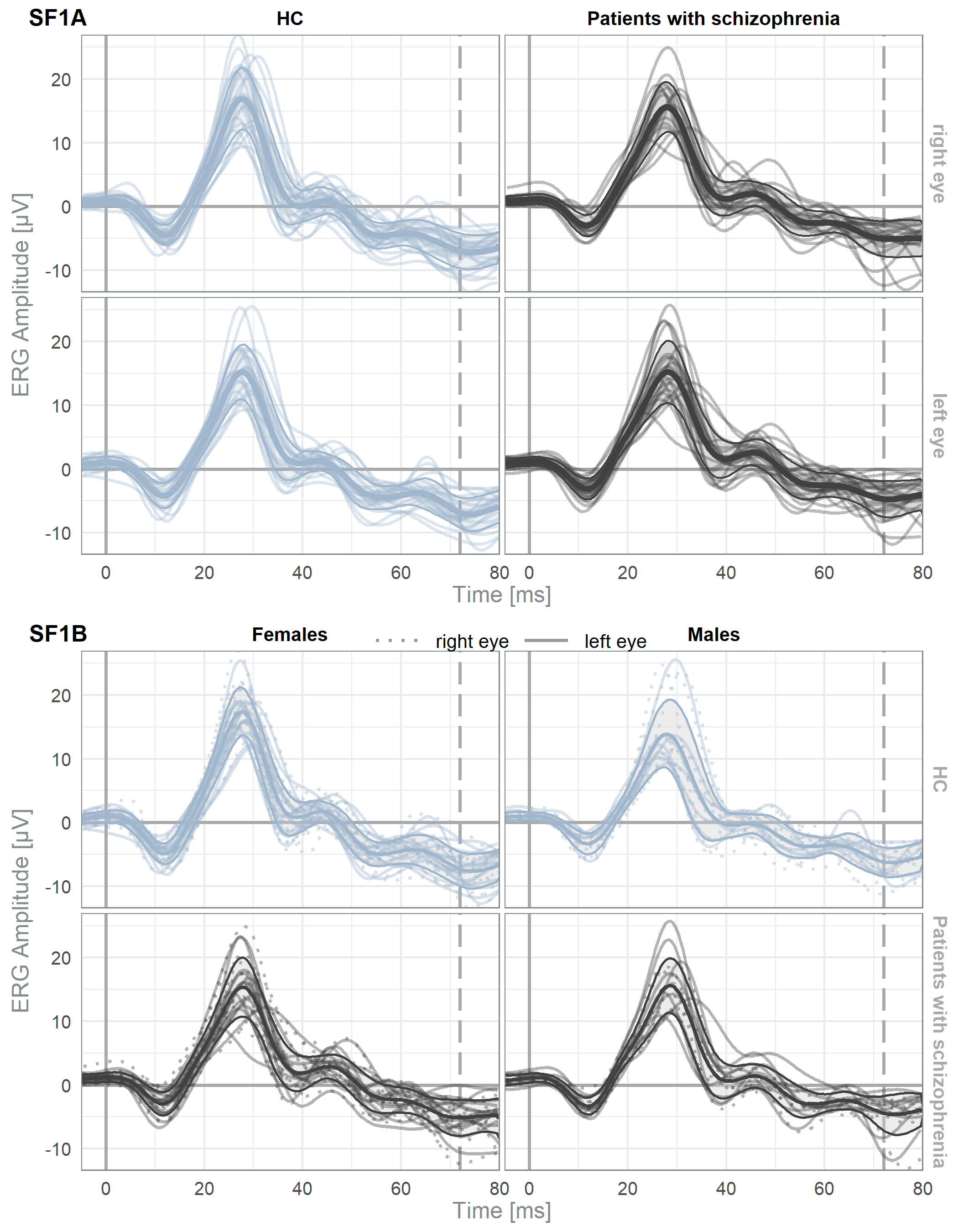

Supplement: Supplementary file 2 — Figure SF1 [file 41398_2022_2167_MOESM2_ESM.tif]

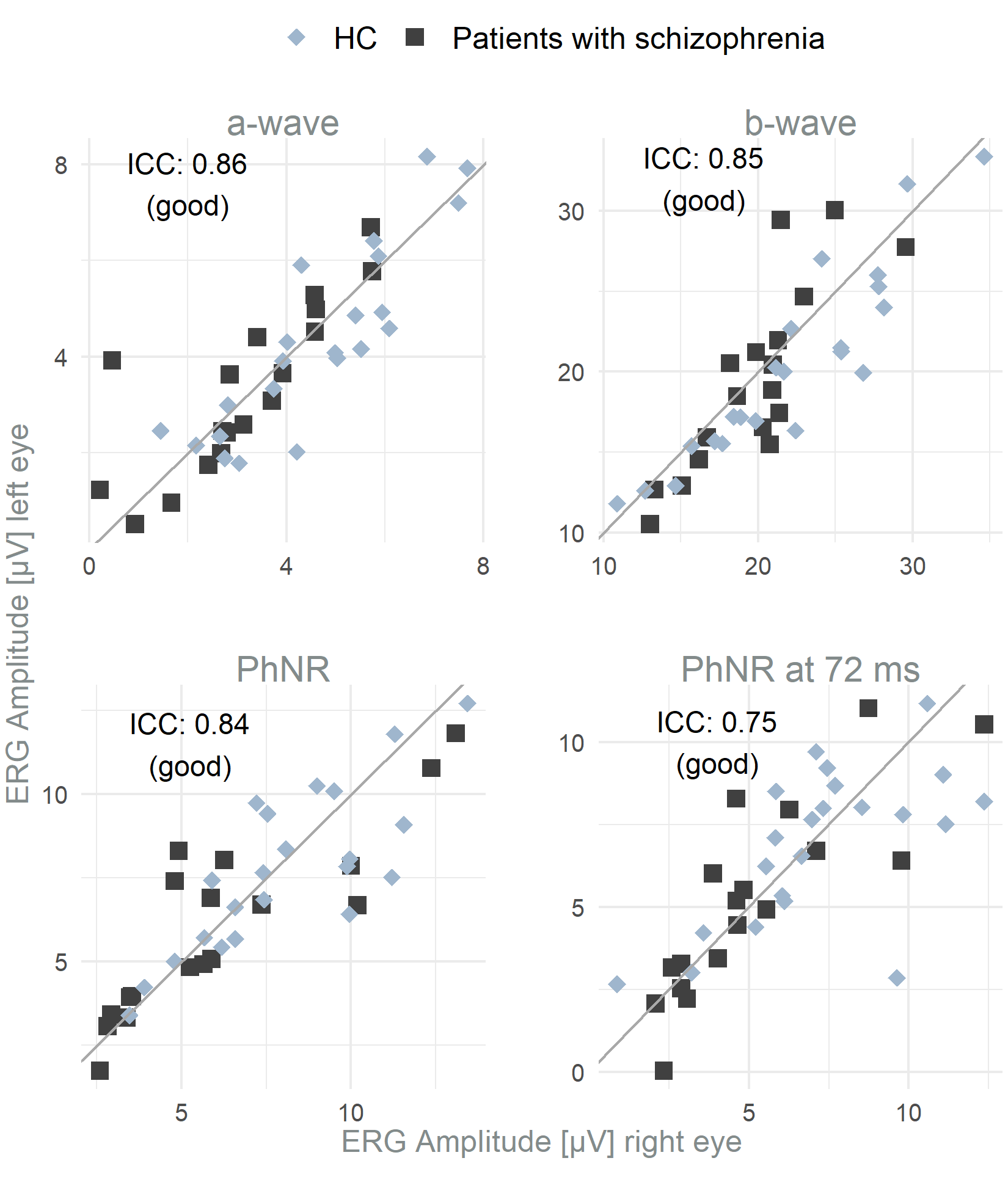

Supplement: Supplementary file 3 — Figure SF2 [file 41398_2022_2167_MOESM3_ESM.tif]
